# St. George's Hospital

**Published:** 1830-10-01

**Authors:** 


### ST. GEORGE'S HOSPITAL.

### Diseases of the Chest.

THE following cases will not, we hope, be devoid of interest at the present time, when diseases of the thoracic viscera are attracting so much and such deserved attention in this country. The advance which has been made within the last half century, in our precise notions of organic changes, is no where more conspicuous than in this class of complaints; for even under the masterly hand of Cullen, they were still involved in that obscurity and confusion which characterise a rude and early stage in the progress of medical knowledge. This may appear a staggering assertion, but we confidently refer to the works of that able master for proof of its correctness. In dedicating a few lines to pericarditis, he observes, that it hardly requires a particular consideration: being almost always a part of the pneumonia he has described: not always distinguished by any different symptoms, or if it be, not requiring any different treatment. The errors of this statement, both of omission and commission, are so flagrant, that we need not go further to demonstrate the great hiatus that exists, between the pathological knowledge of those days and the present.

Unprejudiced persons must allow, that the stride we have taken in this department is mainly owing to the labours of the French, and the cultivation of morbid anatomy. It may be galling perhaps to our national feelings, to be indebted in so great a degree to foreigners, but science, ability, and industry are not the exclusive growths of any clime, nor the only offspring of any institutions. The advantages possessed by the hospital physicians on the continent in the examination of the bodies of the dead, have been so far superior to our own, that we cannot be surprised at the results in favour of the former. Fortunately our opportunities of treading in the same path are no longer limited, and our taste for this species of information has undergone a sensible improvement. At this splendid hospital almost every body is inspected after death;—the exceptions to the rule are so rare, as not to be worth calculation. By insisting on an examination as a matter of course, very little obstruction is thrown in its way by the friends of the deceased, who, on the contrary, are generally anxious for its performance. We believe it has been hinted that the plan is attended with some disadvantages; as patients are constantly admitted in the latter stages of organic maladies, and swell to a seemingly great amount the annual ratio of mortality in the institution. This circumstance has been laid hold of by the malignant knaves that infest the medical press, and twisted and tortured into accusations against the attainments of the medical officers. The charge carries such palpable absurdity on its face, that we are almost ashamed of noticing the maligners. We had rather, ten thousand times, expose ourselves to their devilish enmity, than do what is rumoured to be done at some establishments:—discharge patients whose recovery is hopeless, in order to keep down the number of deaths within their walls. Such a mode of proceeding may square with the dictates of wordly wisdom, but science is the sufferer; and is humanity a gainer?

To render the stores of information which a large hospital contains, beneficial in some degree to those who have not such opportunities of acquiring it, is the proper aim and object of reporting. There is much that can neither be communicated by writing nor by words, but there is likewise much that can; and our business is with the latter. Perhaps there is no point on which the practitioner who sees little of morbid anatomy, requires information so much as on the subject of diseases of the heart and lungs. The change which the introduction of auscultation has made in their investigation is undoubtedly great, and when this means of diagnosis shall come to be more generally practised and *understood* (for it frequently is one without the other,) we anticipate favourable consequences to the public and the profession. One object of the present report is to shew the accuracy which the stethoscope, in most instances, lends to diagnosis.

We propose to detail five cases:—two of empyema, in one of which the operation of paracentesis thoracis was performed—two of fungus hæmatodes of the lungs—and one of pneumo-thorax. We shall commence with the cases of empyema.

### I. EMPYEMA.

CASE 1. *Scarlatina—Empyema—Suppuration in the Hip-joint—Death.*—Christopher Rolfe, a clerk, æt. 26, admitted January 11, 1830, under the care of Dr. Hewett.

Complains of frequent short cough—expectoration scanty, and "cannot be brought up"—dyspnœa—pain under lower third of sternum increased by deep inspiration or cough—cannot lie on *right* side—pain across right loin, not increased by pressure. On the surface of the belly a slight efflorescence like scarlatina—posterior surface of throat, uvula, and tonsils, of vivid red colour and tumid, pulse 133—tongue red and aphthous, but moist—bowels opened by medicine.

Ill since Christmas-day, when he was attacked with all the symptoms of severe cold and cough, of which he has never got rid. Has made great exertions of mind and body during the last week. Pain in the chest has been present for five or six days, dyspnœa rather longer. Has only been confined to the house, and had medical advice for three days—has been purged and bled yesterday to about a pint, with temporary relief to the pain in the chest.

A gargle, infusion of roses and salts, and cupping, if the skin should become warm, were the means prescribed by Dr. Hewett.

On the next day he was examined by the stethoscope and percussion. The right side of the chest, below the third rib, was as dull as lead, upon percussion, but above that rib the sound was more natural. In the part which sounded so dull the respiration was scarcely heard; beneath the clavicle it was *puerile,* and towards the spine it was *bronchial.* Over the middle and lower parts *œgophony* was perfectly distinct. The left side of the chest sounded very sonorously throughout, and the respiration was puerile. On pressing the intercostal spaces in the upper part of the right side, the patient complained of much pain. There could be no hesitation in forming the following diagnosis:

*Considerable effusion from pleurisy in right side of chest, and compression of the lung towards the spine and mediastinum. Left side healthy.*

Fourteen leeches and a blister were immediately applied, and on the 13th the patient was ordered cal. gr. ij. every six hours. On the 16th, being free from pain, the mercury was omitted, and as the strength was much reduced, a light draught with dilute sulphuric acid was ordered. On the 17th the cough was constant, *dry,* and troublesome—there was some little puffiness of the abdomen—the patient *lay upon his back.* The calomel pill was resumed at night, and a blister was applied. On the 19th tr. digital. ♏. v. were added to each draught. On the 20th, the *patient preferred lying on the affected side.* The sacrum beginning to be sore from pressure, was defended by plaster, and on the 1st of February the following is the report: sleep less disturbed by cough, no expectoration—lies always on *right* side, because when he turns on the opposite he instantly begins to cough—right side of chest universally dull on percussion—sweats much at night—gums not affected—pulse 108, feeble, and occasionally intermitting. On the 5th he was ordered five grains of blue pill with ipecacuan and half a grain of opium every night, and a mucilaginous mixture with a little digitalis. His other medicines were omitted.

On the 6th some more leeches and a blister were applied; an abscess formed over the sacrum and was opened, and the patient now began to complain of some tenderness on pressure in the region of the liver, with a little puffiness of the abdomen. There was also a tendency to diarrhœa. *Œgophony* was only perceptible immediately beneath the clavicle and high on the right back; it had vanished from the parts below that level, a sure proof of the increase of the empyema. The acetate of morphia in doses of ¼ of a grain were tried, in order to procure sleep; but although it answered pretty well at first, its operation was not steadily beneficial. To determine to the kidneys, Dr. Hewett gave a trial to the acetate of potash, a diuretic much lauded in these cases by Laennec. From the dose of ʒi. at night, it was gradually increased to ʒiss three times daily, but no diuretic effects could be obtained, a profuse diaphoresis with an urinous smell supplying its place. The tenderness in the hepatic region continued, with the disposition to diarrhœa. On the 9th of March there were a few clear straw-coloured sputa for the first time; the symptoms otherwise were much the same. Antimonial plasters and a blister had been employed since the preceding report, and occasional symptoms were met as they arose.

On the 13th he was suddenly attacked with a roseola-like efflorescence over the surface of the body, head-ache, and some pyrexia. Effervescing draughts were ordered; he rapidly became very low; the breathing grew fearfully hurried and oppressed, and at 12, p.m. of the 15th he expired, his senses remaining perfect to the last moment of existence.

SECTIO CADAVERIS. Body much emaciated.

*Thorax.* Nearly a gallon of purulent fluid in the right pleural cavity—pleuræ themselves coated with thick yellow lymph, arranged on the surface of the costal pleura in a remarkably honey-combed manner. Diapragm on this side thrust downwards by the empyema. Lung compressed to the utmost against the spine and mediastinum, its antero-posterior diameter not exceeding two inches and a half; its lateral diameter very little more than one inch. In structure it was *carnified*, and of blackish grey colour.* Bronchi on this side remarkably small, and soon lost in the impervious lung.

Much serous effusion in the upper lobe of the left lung, and something like recent peripneumonic engorgement in the lower. Mucous membrane of the bronchi congested—some muco-purulent matter in the larger tubes on both sides of the chest.

About two ounces of clear serum in the pericardium.

*Abdomen*. Some flakes of thick yellow lymph on the convex surface of the liver, and adhesions of no ancient date between it and the diaphragm. Liver forced down very low by the empyema. Mesenteric glands a little enlarged.

*Cranium*. Not examined.

In the left hip-joint was a collection of curdy pus, and the head of the femur was partly dislocated on the dorsum ilii.

CASE 2. *Pleurisy—Empyema—Paracentesis Thoracis, &c.—Death.*†

William Smith, a servant, ætatis 23, admitted February 10th, 1830, under the care of Dr. Hewett.

Pain at the scrobiculus cordis and lower part of the left side of the chest, aggravated by cough, and by inspiration, which is imperfect and abruptly checked—pain in the left shoulder—decubitus on the left side—troublesome cough without expectoration—aspect pallid and phthisical. Pulse 108, rather small—skin perspiring much at night—tongue loaded and whitish—bowels opened by medicine.

Caught cold a month ago, and was seized with pain to the left of the ensiform cartilage. He neglected his illness for four days, when he was bled, blistered, &c. and the pain disappeared. A dry cough alone remained, but a fortnight ago this was nearly gone. Ægophony being present in the left side of the chest, a blister was applied, notwithstanding the apparent cure. Five days ago had a relapse preceded by rigor, and has since suffered from the present symptoms. Has not been subject to coughs, nor, according to his own account, is there any phthisis in his family.*

*Leeches—blister—salines with digitalis.*

On examination next day with the stethoscope and percussion, the dull sound was present in the posterior and inferior parts of the left thorax, as high as the inferior angle of the scapula, and ægophony existed to the same extent. It was obvious that there was effusion.

*V.S. stat. ad* ℥viij. *vel.* x. *Hyd. sub. pulv. ant.* āā gr. ij. 6*tis hor.*

He was temporarily relieved by the abstraction of blood, and leeches were applied. Opium was added to the pills, but they griped the patient, and on the 13th, pil. hyd. ℈ss. was given thrice daily in their stead. Leeches were repeated, and shortly followed by a blister. On the 18th the digitalis was omitted, and on the 20th the blue pill, but the gums were not much affected. The cough being very troublesome at night, the acetate of morphia was tried without success. The chief symptoms now were, a very annoying dry cough, most distressing at night—more or less of hectic fever—hurried breathing—

* Carnification of the lung is the result of compression, and differs toto cœlo from hepatization, the consequence of inflammation. For the more detailed explanation of this state, we refer to M. Laennec's treatise.

† This case has appeared in our respected contemporary, the Medical Gazette; as we were in possession of the details quite as early as our brother-editor, we shall be fully acquitted of plagiarism on the occasion.—*Ed.*

* This statement proved eventually to be untrue, a younger brother having died of consumption.

occasional pain in the left side of the chest—and a disposition to looseness of the bowels. On the 3d of March a little straw-coloured matter appeared for the first time in the spitting pot. A blister was applied and the surface dressed with mercurial ointment, and conium was given freely, with some good effects in allaying irritability. He was put on the mistura menthæ acida, a light tonic. On the 15th the sputa were thick, muco-puriform, globular, and in trifling quantity; the weakness was very great. On examining the chest the left side was almost universally dull on percussion, and by measurement was about an inch less in size than the right. He now also began to suffer flying pains in the right side and shoulder.

On the 21st he was examined by Dr. Hewett, who queried *pectoriloquy* between the 2d and 3d ribs on the *right* side. The left side was universally dull on percussion, and the ægophony had mounted upwards. The effusion had increased much on this side.

We need not particularize the items of treatment—suffice it that it consisted in gentle anodynes and moderate support. On the 8th of April the disease might be said to have reached its acmé; the ægophony had mounted to the top of the left thorax—scarcely any respiration was heard in that side, which measured half an inch *more* than the other—the intercostal spaces were on a level with the ribs—the heart was *seen* pulsating to the right of the sternum. With regard to the right side, it was generally resonant, the respiration was puerile, the voice bronchial, and doubtful pectoriloquy existed under the clavicle. The diagnosis entered was:—

*Abundant empyema in left pleural cavity—tubercles in other lung.*

On the 6th of May we find that he had been free from any expectoration for several days; but on the 11th it returned, although trifling in quantity, and in quality the same as before. During this time the dimensions of the left side of the chest perceptibly diminished, and afforded such a gleam of hope that Dr. Hewett deferred proposing an operation. On the 23d, however, a repetition of the measurement shewed an excess of 1 and 3-8ths of an inch in favour of the left side; and the heart was felt, heard, and seen to be pulsating under the left nipple. It was now too obvious that nothing short of paracentesis thoracis afforded the most distant chance to the unfortunate patient. The question of the existence or non-existence of tubercles presented itself to Dr. Hewett's mind. The aspect was phthisical, and the stethoscopic indications supplied by the right side of the chest, were, to say the least of them, suspicious, for doubtful pectoriloquy existed in the upper lobe of the lung. In opposition to these forbidding circumstances, the patient denied the existence of phthisis in his family, and the sputa had never been tubercular. To this point Dr. H. had directed his most anxious attention, and on no one occasion had he observed in the expectoration so much as presumptive evidence of tubercles. As the leaning of justice is, or should be, towards mercy, so the leaning of the medical man should be towards hope; and after maturely considering the arguments on both sides, Dr. Hewett decided on proposing the operation of paracentesis thoracis to his colleagues. Neither being hostile to the measure, it was performed by Mr. Brodie on the 26th of May.

Previous to puncturing the chest, a grooved needle was introduced between the sixth and seventh ribs, on a vertical line descending from the axilla, and some thin purulent matter drawn off. Assurance being thus made doubly sure, the operation was completed in the same spot; about four ounces of purulent whey-like fluid permitted to escape and an elastic tube introduced into the opening, and allowed to remain, its outer extremity having been closed with a plug; by the occasional removal of which, the quantity of fluid discharged might be regulated, and the entrance of the atmospheric air prevented by its replacement.

In the course of the night he suffered for a short time from a little pain in the left side, and was prevented from sleeping by frequent cough. Next morning he was free from any untoward symptoms, and about a pint and a half of matter had escaped since the operation.

Haust. Nitri, c. Tr. Hyosciami. gr. j. 6tis hor.

29th.—About sixteen ounces more fluid have been discharged—in all, about forty ounces. Has a little pain and much tenderness on the left side, aggravated by cough, which is troublesome; pulse small, soft, rapid. Respiration, accompanied with mucous rattle, *is distinctly audible anteriorly and superiorly on the left side.* Slight ægophony below the axilla; dull sound still upon percussion. Heart felt, and heard more distinctly on the left side than on the right.

The flexible tube was withdrawn in the night, as it occasioned uneasiness. No more fluid had escaped, and the entrance of air by its sides, where ulceration was beginning, was apprehended. On the 30th the respirations were 28 per minute; pulse 133, very soft, and feeble; tongue rather furred at the base.

The respiration was audible, but distantly, as far as the inferior angle of the scapula; and the dull sound on percussion had disappeared anteriorly and laterally. The heart pulsated in its right situation; one mass of viscid muco-purulent sputa in twenty-four hours.

He was now ordered moderate support, with wine and sulphate of quinine &c. and from the date of the last report to the 7th of June, there was little alteration for better or worse; the cough was still troublesome, and there was rather more muco-purulent expectoration. On the latter day the respiration was found to be less distant than before; the sound on percussion was a little clearer; and the left side was now an inch less by measurement than the right. On the 8th, the flame of a taper being applied to the wound was not agitated during respiration—a little serum only oozed out. Four or five ounces of muco-purulent matter had been expectorated in the last twenty-four hours. On the 9th, aphthæ appeared on the tongue; and in the morning of the 12th the patient complained of having *suddenly felt air issue with a gurgling noise out of the orifice, whenever he coughed.* Serum also oozed out in abundance; and both were most horribly offensive to the smell, blackening the plaister, and having all the characters of sulphuretted hydrogen. The flame of a taper applied to the wound was driven forcibly away on coughing, but not the least was drawn inward upon inspiration. The left side of the thorax was *preternaturally resonant.*

Next day no respiration was heard on this side, and the metallic tinkling, the pathognomonic sign of pneumo-thorax, was so distinct as to be heard by many persons quite unacquainted with auscultation. At 10, a. m. of the 15th the patient died.

NECROPSIA:—12 *hor. post-mortem.*

Body much emaciated; left side of the chest resonant, like a drum. Prior to opening the thorax, the nozzle of a pair of bellows was inserted into the trachea, and on inflating the lungs air was propelled from the fistulous opening in the left side.

*Thorax.*—About two pints of sero-purulent fluid in the left pleural cavity: this occupied its dependant half; the remainder was occupied by air. Pleura itself, and the costal in particular, extremely vascular and flocculent, lined with false membrane, of varying thickness, and with "concrete pus;" the greater part was in the neighbourhood of the fistulous opening. The lung was much compressed, its structure grey-coloured, and not yet carnified, and a few miliary tubercles existed in it; at the apex was a vomica, scarcely larger than a Barcelona nut, communicating, by a small round opening, (the edges of which were lined with soft lymph,) with the pleural cavity. The vomica again communicated with the superior great division of the left bronchus, by means of a ramification of tolerable size.

Right pleura vascular in parts, and in parts united by adhesions of various dates; no fluid whatever in this side. Its surface both costal, pulmonary, and diaphragmatic, studded with minute, pearl-like, tubercular accretions. Lung itself not collapsing, studded with many soft, common tubercles in its superior lobe, with miliary tubercles in its inferior, but comparatively free from either in its middle lobe. At the apex of the lung several honey-comb vomicæ, of small size, communicating partly with each other.

*Abdomen:* — Several ulcerations of the mucous membrane of the intestinum ilium, near its termination. Ulceration also of the valve of the cæcum, and large patches of the same in the ascending part of the colon. Mesenteric glands enlarged, as they often are in phthisis, rather cheesy in consistence, and in one or two instances suppurating in their centre.

*Cranium:*—Not examined.

The two preceding cases are severally excellent specimens of empyema arising from different causes, or rather in different habits, and running a different course. In the first, it followed an attack of neglected pleurisy, and was totally unconnected with tubercular or other disease of the lung; in the second it was also produced by pleurisy, but the latter was combined with, if not dependent on, tubercular formations. For the modes in which the symptoms differed, and the points on which they agreed, we cannot do better than refer to the details of the cases themselves. We may remark that they shew very clearly the ordinary progress of a case of empyema. A patient gets a cold which he neglects, or an attack of pleurisy for which he is treated, and, if the former, he appears to be recovering: if the latter, to be convalescent. But the calm is delusive, the amendment fallacious; for a certain quantity of fluid has been effused into the cavity of the pleuræ, and the germ of his destruction is there. The symptoms denoting, or rather attending, this critical state appear to be:—a dry and more or less troublesome cough, some slight and flying pains in the affected side and shoulders, and, if the inflammatory action has been subdued, a disposition to lie on the same side of the body. In the first case there was tenderness of the intercostal spaces on pressure, and this is commonly a very valuable symptom of pleurisy. On using percussion the sound will be more or less extensively dull, and on applying the stethoscope, ægophony will be heard for a limited extent. We have said that, if the inflammatory action has been subdued, the patient will be disposed to lie upon the side affected, and we wish to insist on the following rule with regard to the position of the patient, as one of the greatest practical importance, in determining a diagnosis:—so long as acute inflammation of the pleuræ remains, the patient will lie upon the opposite side; but as soon as the inflammation has subsided, or has assumed the chronic character, and effusion to any extent has taken place, he shifts his position and obstinately reclines on the side in which the fluid is placed. The reason is obvious. Whilst active inflammatory action is present, lying on the side affected would be virtually applying pressure to an inflamed organ, and we know that this must be a source of suffering. But when once there is effusion, the latter by its weight would oppress not only the lung on its own side, but also on the other, unless the patient by lying on the same prevented these effects of the gravitation of the liquid.

As the effusion becomes converted into actual empyema, and as the latter increases till it fills the whole of the pleural cavity, the dull sound increases, the respiration becomes fainter and ultimately disappears; the ægophony mounts higher and higher; keeping pace with the progress and marking the border of the still augmenting fluid. At last the whole side of the chest sounds dull; ægophony is scarcely heard, the heart is distorted, the liver may be thrust down, peritoneal inflammation may even occur, hectic fever is set up, and the patient dies with many of the symptoms of phthisis pulmonalis. In the first case we have detailed, the expectoration was scanty to the last, and generally glairy mucus only.

We think the foregoing cases will shew the precision, with which the progress of the malady may be guaged and determined by percussion and auscultation. We do not assert that empyema can never be recognized without them; we know full well that it can, and that it often has been so. But we contend that by their means the diagnosis is more certain when the malady is fully established, and we boldly maintain that, in the earlier stage, it is *impossible* to arrive at any definite conclusions, independent of the assistance they afford. Most persons in practice must have witnessed cases, in which empyema was found after death that was never suspected during life. We have seen such cases ourselves, nay more, we have seen them occur under circumstances, which rendered the chances of success from an operation by no means inconsiderable. We hope that they who may have made such mistakes, and to our personal knowledge the best physicians have done so, will be slow in contemning auscultation.

The only point on which it remains for us to touch is the operation. The first case we have detailed was certainly more favourable for its performance than the latter. The unsuccessful termination in that instance can neither be attributed to the dangers of the operation itself, nor to the empyema for which it was performed. Tubercles and vomicæ were the cause of the patient's death. We have heard that a hospital physician of this metropolis, has met with a success unparalleled hitherto, in the performance of the operation for empyema. It is said that a proportion of seven in nine, or thereabouts, have recovered in consequence of paracentesis thoracis. If this be so, the advancement of science and the interests of humanity demand that the cases should be published, in order that less fortunate operators should learn, and less bold physicians should not dread, the method of affording relief in these desperate cases. It may be prejudice, perhaps, but we fear that report has magnified the benefits derived by the gentleman to whom we have alluded, from paracentesis thoracis; and we look with a sorrowful incredulity on success so incomparably greater than has ever yet been obtained. The only mode of lulling doubt and dispelling scepticism, is to bring the cases before the profession, when all may read, and all may be convinced.

### II. PNEUMO-THORAX.

CASE 3. *Pneumo-thorax — death — tubercles, and small vomicæ in the lungs.* —James Smith, a footman, ætatis 21, admitted March 8, 1830, under Dr. Hewett.

Pain under the inferior half of the sternum, which is not elevated by inspiration— hurried breathing — some cough—no expectoration. Prefers lying on the *left* side, but is obliged to change his position frequently in consequence of the cough—no tenderness of the abdomen, nor pain of the head. Pulse 140, very small—aspect pallid and strumous—bowels freely open.

Has been ill six days, but had a bad cold previously. Was suddenly seized in the morning of the 2nd, with severe pain in the lower part of the *right* side of the chest, for which he was bled to syncope on the following morning, cupped on the 4th, and bled again on the 5th. The pain was relieved by these depletory measures, but not removed, until after a second bleeding on the last mentioned day, since which he has suffered from weakness only. Has had none of the usual symptoms of fever; has been subject to winter cough; his mother died of a decline.

A blister, with calomel and opium every six hours, were prescribed by Dr. Hewett, and afforded a little relief to the cough. On the 10th he was examined with the stethoscope, and the nature of the case rendered but too evident by auscultation and percussion.

The right side of the chest, anteriorly, was preternaturally resonant, from the clavicle to the inferior margin of the false ribs; posteriorly, it was also very resonant, but not to so great a degree. Throughout the right side the respiration was so distant as scarcely to be heard; near the mediastinum, just below the clavicle, and along the spine, it was louder and bronchial. In the latter situation, there was heard a remarkably stridulous, and almost metallic sound, accompanying the *respiration;* the voice gave bronchophony; and although there was no metallic tinkling, there was occasionally the *bourdonnement amphorique.* There could be no hesitation in pronouncing on.—

*Pneumo-thorax of the right side, with liquid effusion the consequence of pleurisy.*

With regard to the other side of the chest: the sound was not equally and universally good on percussion throughout—the respiration was puerile, in some parts even bronchial. These signs when coupled with the history, led to the diagnosis.

*Tubercles in the lungs—no evidence at present of actual vomicæ.**

These facts influenced Dr. Hewett's practice, for being thus assured of the hopeless nature of the case, he avoided calomel and all heroic measures; contenting himself with mild aperients, and effervescing draughts with the tincture of digitalis. The unhappy patient escaped the torture which a well meaning practitioner, not acquainted with auscultation, might have inflicted, in the shape of bolus, mixture, or depletion. If his malady was beyond the reach of art, if it passed the bourne of human skill, the able physician to whose care the case had fallen, was conscious, at least, that he avoided all unnecessary and mischievous interference. A genuine acquaintance with morbid anatomy may not always confer on us the power of curing, but it frequently will prevent us from scientifically killing.

On the 11th, the right side was found to measure 1$\frac{4}{8}$ths inch more than the left; and on the *Hippocratic succussion*, the splashing of a fluid was distinctly audible. No metallic tinkling—pulsation of the heart heard rather farther than natural to the left. Pulse 120—respiration 32 in the minute—lies always on the *left* side—hectic. On the 14th the lowest part of the right side was found to sound more dull, and the tintement metallique was distinctly heard by Dr. Hewett. This shewed that the fluid in the right side was increasing, and proved that the pneumothorax depended on a fistulous communication of the pleural cavity with the bronchia.

Inflammation of the absorbents in the left arm, in consequence of the venesection practised before his admission, now set in, and proved a source of excessive anguish, and no slight detriment to the unfortunate young man. The cough was mostly troublesome, the sputa scanty and muco-puriform, the pulse rapid and rather vibratory. Occasional nausea was complained of, and profuse perspirations took place at night. Small doses of the medicinal hydrocyanic acid were employed with trifling relief to the cough, and it was necessary to have recourse to opiates at night.

On the 1st of April we find that for several days he had suffered from a little pain in the lowest part of the right side of the chest, and the liver was found to be protruded downwards. The dyspnœa was very marked; the aerio-serous effusion two obviously increasing. On the 2nd the respirations were 40 in the minute. Night delirium set in, exhaustion made progress, the patient was harrassed with pain in the right side, and much tenderness of the intercostal spaces on pressure, and early on the 4th of April he expired.

SECTIO CADAVERIS:—2, *p. m.* *5th.* Right side of chest more prominent than the left, particularly in the inferior part; and very resonant, except in the dorsal and inferior regions. Considerable emaciation.

*Thorax.* On puncturing the right side, a stream of air, not at all putrid, escaped. In that pleural cavity was upwards of a quart of green, but not very turbid, sero-purulent fluid. The space occupied by the air was to that occupied by the fluid nearly as 1$\frac{1}{2}$ to 1. On inserting the nozzle of a pair of bellows into the trachea, and endeavouring to inflate the right lung, little, if any, such inflation took place, but air bubbled up through the fluid in the pleural cavity, apparently from an opening in the posterior part of the lung. The pleuræ themselves were coated with "concrete pus," and presented at the apex and base of the cavity a few very

* It must be remembered that these conclusions were arrived at, and expressed, very long prior to the patient's death. The events revealed by dissection, will prove how true were the auscultic indications.

long and firm bands of adhesions. The diaphragm was pushed downwards, the mediastinum deflected towards the left.

The lung was compressed towards the spine and mediastinum, flattened, carnified. The opening through which the air had escaped was situated at the lower part of the middle lobe, very near the spine. It was larger than a pea in diameter, surrounded by soft lymph, and opened into a little vomica, capable of containing a small horse-bean. The latter communicated freely and directly with a large bronchial tube. The bronchi on this side were contracted in diameter.

Pleuræ on left side only shewing some firm adhesions at the upper part. Lung small; its upper lobe sprinkled with a few scattered groups of common tubercles. At the posterior part were two or three vomicæ, the size of small nuts, communicating with each other.

About an ounce and a half of greenish serum in the pericardium, with some flocculi of loose recent lymph.

*Abdomen*:—Some yellow serum in this cavity, with a little recent lymph between the liver aud diaphragm, especially on the right side. Liver pushed down, but healthy, as were all the other abdominal viscera.

*Cranium*:—Not examined.

The left arm, in which the inflammation after bleeding had occurred, shewed serum and lymph in the cellular membrane; a small collection of pus over the external condyle, a minute carious spot on the condyle itself.

This case adds another to the two of pneumo-thorax which we have recorded, and which, as far as we know, are the only instances of the disease having been recognized in this country, during the existence of the patient. The reason is apparent;—without auscultation or percussion, it is physically impossible to ascertain its presence. We have seen two or three examples of the disease in the patients of physicians who did not employ auscultation, but it had not been recognized before death.

The history and progress of these cases are as follows:—A patient of scrofulous or phthisical aspect, labouring under what he calls a cold, perhaps far advanced in phthisis pulmonalis, is suddenly attacked with violent pain in the side. This may be more or less subdued by remedial treatment, but considerable dyspnœa remains, there is a troublesome and nearly dry cough, the patient is disposed to hectic, and can only lie on the opposite side. And observe the difference in this respect between pneumo-thorax and empyema. In the latter, the patient lies on the side affected, for reasons we have attempted to explain, whilst in pneumo-thorax he invariably prefers the other. The reason appears to be obvious: air being lighter than fluid, so long as that is uppermost, comparatively little compression is exercised on the mediastinum or sound lung; whereas, if the patient were to lie on that side, the pressure acting on the thoracic parietes must be communicated nearly undiminished to those parts. We can, indeed, conceive, that when the fluid effusion in pneumo-thorax is considerable, its influence may preponderate over that of the air, and the patient recline as in empyema. Certain it is, that if inflammation attack the other pleuræ or the pericardium, the patient will change his position despite of the pneumo-thorax. Thus, a patient under Dr. Chambers,* having pneumo-thorax, lay as usual on the opposite side; but, being attacked with pleuritis in the latter, he changed his position so long as the acute inflammation lasted, when he instantly resumed his former posture. This happened several times, in successive attacks of pericarditis and pleurisy.

In addition to these general symptoms, which are obviously quite inconclusive in themselves, the patient has the ausculttic symptoms of pneumo-thorax. These we need not specify farther than by saying, that with little or no respiratory murmur, the chest is exceedingly resonant on percussion, and the *bourdonnement amphorique*, or the *tintement metallique* exist, according to circumstances.

---

* Geo. Canning, whose case we detailed in a late Number.

The diagnosis being established, the prognosis is but too unquestionably gloomy. The only possible chance for the patient is from operation, and that can give scarcely a shadow of hope. In the immense majority of the cases of pneumo-thorax, the air has escaped from a tubercle or vomica bursting through the pulmonary pleura. Under such circumstances we operate for pneumo-thorax only to leave phthisis behind, a melancholy prospect for patient and practitioner. Still there is a period and a combination of circumstances, when paracentesis thoracis may not only be strictly justifiable, but absolutely called for. If the effusion has increased to so great a degree that the patient is in danger of suffocation, as was Mr. Cornish, we conceive that the physician must throw these ultimate considerations overboard, and neglect remote evils to administer present relief. The operation is a trifling one *per se*, its immediate consequences are very seldom fatal, the sufferings for which it is performed dreadful, the relief it affords both great and instantaneous. It must be remembered that pneumo-thorax is frequently produced by a tubercle giving way in a *very early* stage of phthisis : a stage which, if not susceptible of cure, is certainly far from being speedily fatal. In the case of Canning, to which we have alluded, there were comparatively few tubercles in the lungs, and in that of Mr. Cornish they were by no means abundant. On the whole, we should say that, in empyema, the operation is much more likely to succeed than in pneumo-thorax; in the former it may be curative—in the latter it is palliative only.

We find that we have not space for the cases of fungus hæmatodes, which we, therefore, defer until our next report. If the present has run to some length, we can only remark, in extenuation, that the subject is become one of extreme importance, and is ill understood in all its bearings by the mass of practitioners in this country. Our object, too, has been to impress on the minds of our readers the practical value of auscultation ; and this being almost in its infancy here, we were necessarily compelled to dilate on points, which, if generally comprehended, would require no explanation.

---

### VIII.

### CLINICAL REVIEW.

### XXXVI.

### ST. GEORGE'S HOSPITAL.

### MALIGNANT DISEASE OF THE LUNGS.

In our last report from this hospital we related two cases of empyema and one of pneumo-thorax, and we promised to complete the series by detailing the particulars of some instances of malignant disease of the lungs. Fungus hæmatodes not unfrequently attacks these organs, but genuine scirrhus is more rare. Of the former we have witnessed numerous examples, of the latter we do not remember one. Fungus hæmatodes of the lungs may occur as a primary or a secondary disease, with an external tumour or without one. It is not uncommon for patients who have submitted to operations for fungus hæmatodes of the extremities, the breast, the testicle, to die at a subsequent period, with sudden symptoms of effusion into the chest; and on examining their bodies, the lungs are found affected with the original disease. Such is the experience of Mr. Brodie. Our present business is not with such cases, our intention is to confine ourselves to fungus hæmatodes or scirrhus, originating spontaneously in the organs of the thorax.

Some slight notices of this affection will be found in the works of Dr. Baillie, and in the observations of Mr. Wardrop on fungus hæmatodes. But they are superficial, and furnish little more than an advertisement, that such a disease does exist in the lungs. As far as we know, the subject has not yet received any material elucidation from the labours of English writers. M. Bayle described it more at large under the designation of 'cancerous phthisis.' His description conveys a fair quantum of information, but theory has thrown a slight shade over his facts, and morbid anatomy has advanced some strides since the days in which he wrote. The most complete, yet succinct, enumeration of the anatomical characters of the disease, will be found in the work of M. Laennec. Our readers should hasten to peruse his chapter on medullary tumour of the lungs, and up to a certain point they will find their curiosity amply gratified. But in that we search in vain for symptoms; like the rods of the Magi of Pharaoh, they are swallowed and absorbed by the Aaron-like wand of morbid anatomy.

Perhaps it will be better before we proceed to individual cases, to mention in a rapid and cursory manner the chief varieties of medullary tumour discovered in the lungs. We will not enter on the history and progress of the morbid growth, as our readers must be fully acquainted with most of the professed treatises on this subject. Laennec then divides medullary tumours into the encysted, unencysted, and diffused. In the first, the tumours are usually numerous, small, enveloped in a cyst of more or less thickness and consistency, and commonly divided by firm cellular tissue into several lobes. The second, or *unencysted*, are frequent, varying in size from a hemp-seed to that of a full grown fœtal head, spheroid or irregular in shape, lobulated, invested with cellular membrane of greater or less consistence according to circumstances; they are most frequent in the limbs and larger internal cavities. The third variety, or the *diffused*, in which the organ or organs is infiltrated with the medullary matter has never been seen by M. Laennec in the lungs, nor have we observed it in any part of the body. We therefore pass it by without further notice. In concluding this short exposé, we may remark that the distinction between encysted and unencysted medullary tumour is often insusceptible of close discrimination. Most of the medullary growths in the lungs are enveloped in a thin cyst, in their earlier stages, and in proportion as they advance the boundaries grow less defined on the one hand, or more perfect on the other. When the lung is becoming more and more implicated in the malady, the line of demarcation is feeble, is lost; where chance or the processes of nature have arrested its march, lymph deposited around forms a bolder barrier.

Another important point remains. Whatever be the kind of medullary tumour, three well-marked stages are noticeable in its progress. In the first, the depositions are small, and white, and intersected with few blood-vessels; the constitution seems to sympathise little, the nature of the malady cannot be suspected. In the second, the morbid growths have advanced in size, their fine pearl-like colour is somewhat dimmed, the centre shows a disposition to soften, large blood-vessels run from the neighbouring parts to the surface, and others of some magnitude display a partial or universal net-work in the centre; now is the time for small extravasations of blood to take place in the tumours; for the constitution to sympathize; and for symptoms to be set up. In the third stage, the tumours have spread rapidly and widely, and softened as they spread: the extravasations of blood are numerous, and often so extensive as almost to destroy the medullary character of the disease; the patient is evidently wearing away with pain and irritation and profuse sweats, and at length he either dies exhausted, or is suddenly cut off by effusion on the other lung.

With these preliminary observations we proceed to the report of individual cases.

### FUNGUS HÆMATODES.

CASE 1. *Contraction and subsequent Enlargement of Left Thorax—Sudden Symptoms—Death—Fungus Hæmatodes of Left Lung.*

Benjamin Long, æt. 27, ostler, admitted Dec. 16th, 1829, under the care of Dr. Hewett.

Cough—sensation whilst coughing of a knife cutting him in left side—decubitus on that side—inspiration very imperfect and wheezing—uneasiness in the throat—emaciation—aspect phthisical. Pulse very frequent and weak—skin cool. Sweats at night.

Attacked six weeks ago with cough, dyspnœa, sharp cutting pain in the left side sometimes extending to the right, and frequent chills succeeded by fever and thirst. For the first fortnight he spat very small quantities of blood, of a scarlet colour. After three weeks of illness he convalesced; but a fortnight ago he experienced a relapse, and has since suffered from the present symptoms; the night-sweats have been present for a week. Was previously a healthy man—has not been bled for this attack. *A blister—salines with digitalis, &c.—and mild sedatives.*

On the 20th a more precise examination of the case was had recourse to. The pain in the side had been diminished by the operation of the blister—he suffered from a rather troublesome cough—*the sputa were very scanty, and merely transparent mucus, or rather glairy, frothy fluid*—the pulse was small, frequent, soft—and he was disposed to attacks of vomiting.

The left side was less expansible than the right, and also measured less; the respiration on this side was inaudible; the sound universally dull. There was no ægophony. The right side was very sonorous, and the respiration was puerile.

*It was supposed that the left lung was consolidated, and the pleuræ united after pleuro-pneumony. It was evidently a case of contraction of that side.*

We need not specify the particulars of the treatment: it consisted in attention to the bowels, and quiescent remedies. Physic could not remove an organic change, and violent measures were carefully avoided by Dr. Hewett. On the 4th of January, the patient thought he had caught fresh cold, for the cough was more annoying, but the sputa remained as before. He had also pyrosis, for which alkalies were ordered. The bowels being irregular, sometimes costive, sometimes too relaxed, and the febrile action becoming more prominent, some blue pill with digitalis and opium were given every night for three or four weeks, without in the least affecting the mouth. During this time the prominent symptoms were the cough, nocturnal perspirations, scanty, transparent and glairy sputa, and occasional sense of "tightness" at the chest, for which leeches were applied. On the 14th a slight streak of florid blood was noticed in the sputa. The action of the heart now attracted attention; it was tumultuous and appeared extensive. On applying the stethoscope, no *bruit* nor increase of impulsion were perceived, but the sounds were louder and heard more extensively on the left side than usual. The organ was thought to be somewhat dilated. On measurement the left side was one inch less than the right.

On the 13th of February, a few sanguineous striæ were again observed in the transparent sputa; the *right* side now sounded unequally beneath the clavicle on percussion, and about the second or third rib pectoriloquy was thought to exist. It was queried:—*Tubercles in upper part of right lung—a small vomica?* On the 27th he complained for the first time of "flying rheumatic pains in the right side of the chest and shoulder." Volatile liniments were applied, but on the 3d of March the pain was found to be more severe; the sputa were more copious and opaque and contained some blood; the hectic was well-marked. Leeches to the side with demulcents were the means employed.

On the 24th both sides of the chest were found to be of *similar dimensions:* consequently the left had gained an inch within the last few weeks.

In the morning of the 30th he was seized with vomiting of glairy mucus, and at 1, p.m. he had a rigor, followed by slight pyrexia, and pain in the head. Effervescing draughts, and a mustard sinapism to the epigastrium were ordered. On the 1st of April the vomiting continued, with pain in the stomach, small and frequent pulse, anxious countenance, and slight delirium. A blister to the epigastrium—effervescing draughts, with the liquor opii sedativus. By these means the vomiting was partially relieved, but in the evening there supervened severe pain in the left side of the chest, increased by motion or pressure; little cough—no expectoration. The left side now *measured ¾ of an inch more than the right*—ægophony was heard below the axilla and over the scapula.

*It was clear that effusion had taken place between the left pleuræ.*

Early in the morning of the 3d the patient died.

*Sectio Cadaveris; 28 hor. post mortem.* Body emaciated, but not so much so as in phthisis pulmonalis.

*Thorax.* Some old cellular adhesions between the pleuræ on right side. Upper lobe of right lung presenting some scattered groups of miliary tubercles. In anterior part of lung, immediately below the pleura, and about two inches from the apex, a single vomica, somewhat larger than a nut.

Pleuræ on left side, united by old adhesions in various parts anteriorly; very firmly adherent posteriorly, and inferiorly joined by cellular adhesions, into and between which a pint or more of recent bloody serum, with a few flakes of lymph, had been effused.

Left lung irrespirable throughout; its volume rather diminished than increased. It was almost universally occupied with masses of fungus hæmatodes, not encysted. They varied in size, were irregular in form, and generally of whitish colour. Some were slightly softened, others very much so, and the centre always shewed this state the best. In some parts the distinctive medullary character had disappeared, the texture of the lung was in great measure converted into a puriform, putrid-looking bouillie, and the whole bore a certain resemblance to the diffuse suppuration of this organ; it differed from it, however, in many material points. The most disorganized portions of the lung were the inferior and posterior; at its summit was a trace of mere carnification.

Between the pleura pericardiaca pulmonalis, and diaphragmatica, was a mass of rather fatty-looking matter, which appeared to have been deposited *in the cellular adhesions* between the pleuræ. Vessels passed to it from the latter.

Between the great bronchi, pressing on the œsophagus and the aorta was a separate mass of fungous matter, chiefly consisting of the disorganized bronchial glands. The great left bronchus was surrounded by it, encroached on, and amalgamated to it in such a manner, that the cartilaginous rings could no longer be felt, and the diameter of the tube was exceedingly diminished.

Considerable quantity of serum with flakes of lymph in the pericardium, which was more opaque than natural. At the angle of reflexion between the loose and adherent pericardium, opposite the left auricle, one of the medullary masses protruded in some measure into the pericardiac cavity. The membrane was not destroyed but appeared, though thinned, to be still reflected over the morbid growth.

Heart and great vessels nearly natural.

*Abdomen.* Viscera healthy. In the lesser omentum, a small suspicious-looking mass.

*Cranium.* Not examined.

We shall make but a few remarks on the present case, and pass as quickly as possible to the next. If our readers will peruse the details with attention, they cannot but be struck with the remarkable precision conferred by the stethoscope on the diagnosis. Of course the cylinder could not lead one to pronounce that the disease was fungus hæmatodes ; to do that, it would require an instrument of sight as well as hearing. But it shewed that the whole of the left lung was irrespirable and consolidated—that the pleuræ in the first instance were united, and subsequently separated by sudden effusion—and it led to the suspicion, if it did not prove the existence, of tubercles and a vomica in the other lung. The diagnosis was nearly as correct as human diagnosis can be, and they who affect to laugh at percussion and auscultation would do well to form as accurate opinions. But no, it cannot be ! In spite of prejudice, in spite of learning, in the face of neglect, and in the teeth of opposition, auscultation will prevail ; for truth, like freedom, is ever victorious.

Its battle once begun,

Bequeath'd, like it, from sire to son,

Tho' baffled oft, is ever won.

Case 2. *Large Tumour on the Right Side of the Chest—Absorption of some Ribs—Fungus Hæmatodes of the Lungs.*

James Rickards, æt. 25, a gardener from Aylesbury, admitted April 28th, 1830, under the care of Mr. Brodie.

Nearly globular tumour on the right side of chest, extending from the lower margin of the 4th rib to the 11th, and from near the angle of the ribs posteriorly to within an inch of the cartilages anteriorly—generally firm and elastic—giving a sense of indistinct, deep fluctuation—immoveable on the ribs—rather tender on pressure—and affected with deep-seated pain chiefly felt at nights. Integuments healthy. Right side of chest imperfectly expanded on inspiration, which produces pain at the lower border of the tumour—decubitus on the back only—no orthopnœa—some cough. Pulse frequent, rather wiry—perspires at nights—aspect scrofulous. Right side of chest anteriorly, above 5th rib, extremely sonorous on percussion; below that, perfectly dull—nearly whole of right back the same. Respiration is sonorous, part puerile—below the 5th rib is speedily lost altogether. Indistinct ægophony about 6th rib anteriorly—very shrill broncophonie in right axilla.

About ten years ago strained himself at cricket, since which time he has always experienced tenderness in the site of the present tumour. This he first observed seventeen or eighteen months ago ; it was then no larger than a nut. Two or three months after its first appearance he suffered from a violent, but short, attack of pain in the right side of the chest, and about three weeks ago he had another, which was much relieved by a blister. The tumour has increased with additional rapidity during the last few weeks. Has had cough for about a month—has emaciated considerably.

No doubt was entertained of the malignant nature of the tumour, and the evidence obtained from percussion and auscultation declared, but too unerringly, the implication of the lung below the 5th rib, or at least the extension of the disease within the chest in that situation. The bowels were attended to, mild sedatives exhibited, and leeches applied when the pain became severe.

On the 5th, the tumour was punctured by Mr. Brodie, with a fine grooved exploring needle, but, as had been anticipated, no fluid made its exit. On the 6th, the expectoration, which was extremely scanty and merely frothy, was tinged with a streak or two of florid blood. On the 14th the tumour was found to be increasing at its anterior part, and this was the seat of pricking pain, not aggravated by inspiration. He was ordered bark with tincture of iodine, and shortly afterwards iodine ointment was rubbed upon the tumour. On the 23d he had a fresh attack of severe pain in the situation of the right false ribs, attended with anxiety, dyspnœa, and inability of lying on either side. There was no expectoration whatever. The bark and iodine were omitted, and leeches were applied. On the 27th there appeared, for the first time, pain in the left side of the chest ; there was troublesome cough ; scanty expectoration slightly streaked with florid blood ; the dulness on percussion had mounted higher on the right side, and its expansibility was perceptibly diminished. It was evident that the disease was advancing in the right lung or pleural cavity. Cupping, a blister, salines with antimony, and sedatives procured a temporary respite.

On the 15th of June we again examined the patient with attention. The tumour had decidedly increased in size, especially on the back—it was softer, but gave not the genuine sensation of fluctuation—the cutaneous veins were enlarging. He had violent pain from time to time in the tumour and right side of the chest, the lower half of which was manifestly bulged out. The breathing was hurried and laborious—he had hectic, and copious perspirations. The *whole* of the right side was now dull on percussion, and the respiration was only audible below the clavicle, where the voice gave bronchophony. The sound on the *left* side was now become unequal, and the respiration was generally puerile.

*Nearly whole of right thorax occupied by tumour. Tubercles or malignant deposites in left lung.*

From this time matters went on from bad to worse—the attacks of pain in the chest were more violent, more frequent, and more prolonged—the dyspnœa grew more distressing—he neither lay down by night nor by day, but could only avoid suffocation by sitting up in bed with his head on his knees, and inclined to the left side—the respiration in the left thorax became more imperfect, and attended with a râle—the heart acted jarringly and tumultuously, so that it was thought to be suffering from chronic inflammation—the prostration of strength was beyond description—and, at length, on the 27th of June, death put a period to the poor fellow's sufferings.

*Sectio Cadaveris.* Emaciation—body remarkably bloodless—right side of chest universally dull on percussion—left partially so. Tumour of very great size.

*Tumour and Thorax.* Tumour covered posteriorly by the serratus magnus and latissimus dorsi muscles—anteriorly, only by the integuments. The tumour was inclosed in a thin cyst. The eighth and ninth ribs were separated from each other for nearly three inches about their centres. Between three and four inches from their sternal ends these ribs had been absorbed, and were totally wanting for a space of three inches; their ends were rough and jagged. The ribs above and below were entire but deflected by the growth and increase of the tumour. Through the space occasioned by the absorption of the eighth and ninth ribs, the tumour passed into the chest, and appeared to occupy nearly the whole of the right side, and to pass across to midway between the sternal articulations of the ribs on the left side, and the junction of their bones and cartilages. The heart was deflected to the left and partly overlapped by a process of the tumour.

The tumour itself was lobulated, and on section shewed a perfect specimen of fungus hæmatodes. In parts were rounded masses of medullary matter, in others black recent blood, and in others again dun-coloured lamellated coagula. The upper lobe of the lung was least pressed on, but its texture was inflamed, and contained a number of round, separate, medullary tubercles, mostly of small size. The lower lobes appeared to be deflected towards the mediastinum, and either amalgamated with the tumour, or else so converted into matter of a similar description as to baffle all accurate research. The medullary lobules encroached on the cavity of the pericardium but had not produced inflammation of that membrane.

Left lung studded with innumerable medullary tubera, varying in size from that of a large pea to double or treble the dimensions of a marble. They were all white and cerebriform, without coagula, except to a small extent in two or three. No fluid in either side.

*Abdomen.* The diaphragm protruded downwards, with a little lymph on the convex surface of the liver, and some bloody serum in the peritoneal cavity. The tumour appeared to send a process downwards by the side of the aorta, but this part of the examination was so hurried, that the fact was not fully ascertained. Liver itself free from disease.

*Cranium.* Not examined.

We might multiply cases of this disease were it necessary, but the two already detailed are sufficient for our purpose. They show the obscure symptoms attending its origin and progress, for in the first case there was nothing to lead the physician to suspect the existence of fungus hæmatodes, and in the second it was only revealed by the characteristic external tumour. The history of the affection appears to be this. A man of middle age, or generally younger than that, is attacked with what appears to be inflammation of the chest, perhaps accompanied with slight hæmoptysis. From this he recovers to a certain extent, but his health and strength are not what they were before. A permanent degree of dyspnœa, with hectic, more or less complete, remain behind, and on examination of the chest one side is found to be manifestly defective in the exercise of its functions. There is also cough, but not to a great extent; expectoration of frothy mucus, or muco-purulent matter, trifling in amount, and occasionally streaked with a little blood; emaciation, and night sweats. It must be owned that these symptoms are far from decisive, and we find in practice that they prove so. In fact, unless an external tumour or fortuitous circumstances conspire to indicate the nature of the disease, it is hardly possible for the ablest practitioner to do more than guess at its existence.

The obscurity that wraps the diagnosis is not always dispelled with the progress of the malady. It might be thought à priori that the character of the sputa would supply an indication. In general it does not, and indeed it is surprising that fungus hæmatodes should proceed to so great an extent in the lungs, without communicating to the expectoration any traces of medullary matter. In the first case we have detailed, many parts of the lung were no better than a putrid bouillie, and the bronchus was converted into the disease, yet the sputa were no more than frothy mucus. In some cases, however, the expectoration is excessively offensive. A patient affected with this disease was thought to be labouring under phthisis pulmonalis. Dr. Hewett was struck with the fœtor of the breath and of the sputa, and inferred from those circumstances the existence of something more than common consumption. The event amply verified the justice of the suspicion.

For the later stages and the final result we can scarcely do better than refer to the cases themselves. There is certainly some similarity between the symptoms of this disease and empyema. The same description of cough, the same scanty sputa, the same emaciation, the same hectic, conspire to maintain the accuracy of the parallel, not seldom to deceive the medical attendants. But the history of the cases is somewhat different, and the signs supplied by auscultation are quite so. In empyema, an acute attack of pleurisy is followed by ægophony, and the latter mounts with the progress of the fluid. In fungus hæmatodes, ægophony, as far as we have seen, is absent. There may be cases, however, in which some fluid is effused between the pleuræ, and ægophony thereby produced. This happened towards the close of the existence of Long, the patient whose case we related first. On the whole then, we must return to the position from which we started, that the diagnosis of this disease is frequently involved in impenetrable obscurity, till the knot is cut by the scalpel of the dissector.

For the termination of these melancholy cases, we have just referred to the facts themselves; but, before we conclude, we may venture a few observations on the subject. The patient continues to suffer from hectic, emaciation, slight but teasing cough, and scanty expectoration occasionally streaked with blood. He has evidently some fatal malady preying on the constitution, and sapping the respiratory system. From time to time an exacerbation of the symptoms attacks him. The dyspnœa and cough are more distressing; the pains in the chest are more severe, if they previously existed, or, if hitherto absent, they now become established; the surface is bathed in a dewy sweat; and a deep-seated anxiety is settled on the countenance. In one of these attacks, more violent or more protracted than the rest, the unfortunate patient is cut off. We should say that the "blindness to the future wisely given," so prominent in phthisis pulmonalis, is much less marked in this disease. We might add some more reflections, but we fear that our readers will deem the foregoing more than enough. Our apology is, that they are practical deductions, and not theoretical speculations —the result of what we have actually seen, and not the wild offspring of what we have thought.

Before we conclude we shall add the short particulars of a case of true scirrhus of the lung, communicated to Dr. Johnson by Mr. Calley, of Burton Crescent. The patient was under their joint care. Perhaps more precise details might be desirable, but it is hard to expect the minuteness and precision of a reporter from a gentleman engaged in the hurry and fatigues of practice. The preparation is in the hands of Mr. Davis, and we again repeat that it shews a genuine scirrhus of the lung.

*Case.* Henry Jennings, 15 years of age, of delicate fibre, consulted me on the 6th January, 1830, with rheumatic pain in the right hip. There was very little febrile action; the pulse small—no rigors—a great deal of lassitude with slight cough—no difficulty of breathing, and could make a full inspiration without pain. There were frequent metastases from the hip to the knee and ancle: He was confined until the 5th of February, at which time he was recovered sufficiently to return to his occupation in the city.

On the 1st of April he came to me again, complaining that his cough was troublesome at night, and as he was of delicate constitution, I recommended his removal into the country for a short time. On his return on the 25th of the same month, I observed a great change in him; he had great oppression of breathing, and was obliged to maintain an erect posture; the chest elevated on the left side, measuring two inches larger than the other—could only lay on that side.

Dr. Johnson was consulted the following day, and stated his opinion and conviction that considerable effusion had taken place in the chest, accompanied by organic disease. He prolonged a miserable existence until the 1st of July when he expired.

*Dissection.* On raising the sternum the left lung was found to be almost entirely converted into a whitish mass resembling very firm brain or rather scirrhus, weighing nearly two pounds, and firmly adherent to the sternum. There were five or six pints of clear water in the left cavity of the chest. The heart was rather smaller than usual —the lungs on the right side sound.

*General Remarks.* He was subject to fits for some time until he attained his sixth year, after which there had been nothing remarkable about him up to the time he had the rheumatic attack. He had been observed these two years past, when sitting and not particularly engaged, to droop his head, and when desired by his friends to hold it up he would often say it gave him pain; and although naturally an active cheerful lad, he often complained of weariness and languor; I understand also that he has had a cough these two Winters, with frequent chilliness.

### ST. GEORGE'S HOSPITAL.

### INJURIES OF THE HEAD.

THERE is scarcely a more interesting subject to the surgeon, whether he be of a purely practical turn, or inclined to indulge in the speculations of physiology, than that of the injuries to which the cranium and its contents are exposed. Like all the other great points of surgery, it has emerged from the gloom, which the comparative ignorance of the earlier ages of physic threw around its accurate and sober investigation. The labours of Le Dran, of the French Academicians, and particularly of Mr. Pott, were of essential service in pointing out the proper methods of study, and in explaining symptoms which were previously shrouded in darkness and in mystery. The peculiar talents of Mr. Pott substituted, indeed, a fallacious light for the former shadow; but this has been sobered down by the practical and eminent men of our own times, by Abernethy, Cooper, and Brodie. We do not pretend that the knowledge and treatment of injuries of the head have been reduced to rules of unerring certainty, or deny that much difficulty and doubt impend over many of these cases. But we are confident that the treatment is generally based upon rational, consistent, and intelligible principles, and that a great deal has been done, if more remains to do. The road to knowledge in medicine and surgery is only through the medium of cases. The general notices of facts which we find in essays and books, are rather calculated to illustrate doctrinal points, than convey any accurate information in themselves; and, as an able writer has observed, they admit of the bias of theory and the warp of system, with more facility than cases more closely and circumstantially related. Thus we perceive the value of hospital reports. If taken with care and detailed with fidelity, they are calculated to prove beneficial to the public; but if the source is poisoned, if the reporter sin through malice or incompetence, the profession are quaffing, like Darius, not a wholesome beverage, but a foul, muddy, and polluted draught. Under the best of circumstances, errors will creep in, and men who would not willingly deceive, have nevertheless, through the fallacies of the human mind, and the weaknesses that vanquish the wisest, proved the unconscious instruments of deception. This alone, independent of the prevalent false faith of these days, will always furnish false facts in abundance, for theorists to stumble, rogues to cheat, and honest men to grieve.

It must be allowed that written cases, however minutely observed and carefully recorded, afford an inadequate and imperfect representation of what actually occurs in practice. It is a defect inseparable from this method of communicating information, and although it may be partly remedied by the care and attention of the narrator, it can never be altogether obviated. We must take reports, then, for what they are worth, and after we have allowed for all their imperfections, enough of good and usefulness remains to render them excellent media for diffusing knowledge.

We shall dedicate the present report to cases of concussion, uncombined with perceptible fracture.

CONCUSSION.

Perhaps the simplest case of injury of the head is that in which a person receives a blow upon the part, is stunned for a short space of time, and after his recovery from the state of insensibility, experiences no farther symptoms of consequence. There is much variation in one particular symptom which has often been alluded to—the non-remembrance of the mode and time in which the accident occurred. An individual suffering what appears to have been a trifling injury will present this loss of memory, whilst another, who has seemingly been hurt more severely, will not. As a general rule, the more acute the injury and the more complete the state of concussion, the more perfect is the oblivion respecting the accident.

Some authors have stated that the pupil is dilated in concussion. It frequently is so, but not always. We have seen it contracted, and it varies in a short space of time in the same individual, being contracted and dilated in the course of a few hours. It is almost always sluggish, but in some few cases it would seem to be little, if at all affected, especially if the patient is recovering from the state of insensibility.

CASE 1. *Concussion—Pain in the Head.*

John Nevinton, ætatis 35, admitted July 19th, 1828.

Was knocked down when rather intoxicated, and his head struck the ground. In a quarter of an hour afterwards was admitted in a state of insensibility—pulse low and faint—skin cold —pupils dilated! He had been bled in this condition by a chemist.

In two hours after his admission he was sensible, and able to reply to questions. The pulse had risen to 80, and was full; he complained of pain in the forehead; had no recollection of the occurrence of the accident. He had house physic, and in the evening, the pulse being fuller and the pain in the head more severe, he was bled to ℥xij.

He was relieved by the bleeding, but continued drowsy, although roused without difficulty. The pulse was full and slow. On the 23d there was much uncomfortableness about the head—pulse full, sharp, and remarkably slow—pupils rather sluggish. On the 24th, he complained of dull, aching pain across the brow—the pulse was slow, and presented a trifling irregularity. He was bled to ℥xvj. and took salts in infusion of roses. He was relieved. On the 28th he was ordered a little ammonia, but it occasioned more pain in the head, which he compared to the skull "being opened and shut," accompanied with throbbing in the brow; the pulse was 50, rather vibratory. He was again put on salts and salines; on the 8th, leeches were applied behind the ears; and, on the 11th, a blister was placed behind the left. The pain in the head gradually diminished—it subsided; and on the 18th of August he was discharged cured.

CASE 2. *Concussion—puffy Tumour of Scalp simulating depressed Bone.*

Eliza Macpherson, ætat. 5, admitted Dec. 16th, 1828, under Mr. Brodie.

This little girl experienced a fall upon her head on the 8th of December, by which she was stunned, and after which she vomited. She then recovered her senses, and was brought to the hospital on the 16th on account of a swelling of the scalp, which the surgeon told the mother would require at least half a dozen incisions.

This formidable tumour requiring such active surgical treatment, was a fluctuating swelling extending over the greater part of the left side of the occiput, without any pain in the part or in the head, without any redness of the skin or marks of inflammation in the cellular membrane, and finally, without a single symptom of general disturbance. It was evidently a case of effusion of blood, remaining fluid beneath the scalp. Near the left sagittal suture was a depression, which might well have been considered as produced by fracture. It depended on effusion into the soft parts around.

The treatment consisted in the application of spirit lotion, and one or two aperient powders. It is needless to say that no incisions were made, and on the 23d the swelling, fluctuation, and appearance of depression were all gone together, and the child was as willing and able to run home as if nothing whatever had happened to her.

CASE 3. *Slight Concussion—Puffy Tumour of Scalp.*

Joseph Drew, æt. 26, admitted June 12th, 1830, under Mr. Babington.

On the left side of the head, about two inches above the ear, was a circumscribed hard tumefaction, exceeding a crown-piece in circumference, with a soft and boggy kind of depression in the centre—not much tenderness on pressure—no loss of sense—pulse quiet. Fracture of the lower end of the radius.

Had fallen in the morning from a hay-loft, seven or eight feet from the ground. Was stunned for ten minutes, but did not vomit. The case was clearly one of puffy tumour of the scalp.

We need scarcely mention the treatment; suffice it to say, that on the third day the tumour had disappeared.

CASE 4. *Severe Concussion—Puffy Tumour of Scalp—Bleeding from the Nose and Mouth.*

John Smith, a groom, æt. 28, admitted June 25th, 1830, under Mr. Brodie.

At half-past ten o'clock this morning a horse with a gig ran away, and both passed over him. He was stunned for a short time, and when carried home was immediately bled to nearly a pint, when he vomited. Blood issued from his nose and mouth in considerable quantity.

*Half-past* 11, *a. m.* Great ecchymosis about the left eye and left half of forehead—sensation, communicated by the effusion of a depression on the latter—left clavicle broken, with various other bruises. He is partially insensible, but answers questions if long and loudly hollaed to—moans much—surface cool—pulse feeble.

After his admission he vomited two or three ounces of dark blood, and in the evening he vomited some more. His symptoms continued the same.

On the next morning, his pulse having risen, he was bled to ℥xij. and one of the cups shewed the usual inflammatory appearances in the blood, but the other did not. The pulse was from 70 to 80, full, the tongue dry and brown in the centre. The pupils were contracted, but contractile—he was constantly moaning—could answer questions when aroused—but did not know where he was, nor how nor where the accident had happened.

*Lot. frig. capiti.—H. sennæ.*

On the 27th he was much improved, and the effusion of blood beneath the scalp was less apparent; the peculiar depression was almost gone. He had vomited no more blood. The fracture of the clavicle was attended to, and although he complained for some time of pain in his head, and was very unmanageable, the unfavourable symptoms gradually passed away, the clavicle united with a little riding, and on the 21st of July the patient was discharged from the hospital cured.

CASE 5. *Severe Concussion—Inflammation of the Absorbents of the Leg.*

Thomas Fox, æt. 11, admitted April 16th, 1830, under Mr. Keate.

*One, p. m.* Was thrown from a horse at full trot, an hour and a half ago, and is said to have fallen backwards and struck the occiput. Was picked up in a state of insensibility, and has so remained; has vomited several times, and has been bled to ℥viij.

He lies coiled up in bed as if asleep—is unconscious of what is said to him, but shrinks when pinched, and resists all attempts to move him. Pupils neither dilated nor contracted, but sluggish—pulse slow and feeble—respiration natural.

*Calomel and black draught.*

In the evening he could give peevish answers to questions when sharply put to him, and next morning was more sensible still, asking for the chamber-pot, and complaining of pain in the forehead. Pulse 60, soft, occasionally irregular—pupils sluggish, and more dilated—bowels freely opened.

*H. sennæ. Hirud.* x. *pone aures. H. sal. c. vin. ant. t.* ♏. xv., *mag. sulph.* ℥j. 6*tis hor.*

On the 28th there was little change; the pulse was 56. On the 29th he lay like a child with hydrocephalus—the pupils were *contracted,* yet the eyes were open and staring—no stertor—hand often carried to the head—no replies made to questions—pulse ranging from 56 to 60—bowels open under him. Next day he continued in the same state; the pulse was 56, and had been 48 in the morning; herpes had appeared about the lips and chin.

*Emp. Canth. nuch. et servetur ope. Ung. Hyd. fort.*

On the 1st May he was more sensible, and could put out his tongue, which was moist—pulse 48—motions no longer passed beneath him. He was ordered support in the shape of beef-tea.

In the evening of the 2d, he was feverish and heated, and less sensible. Pulse 56—tongue white, coated—pupils dilated — motions passed in bed. On the next day, being still in the same state, he was bled in the morning to three ozs., and again in the afternoon to five. The mouth was now rather mercurialized; he complained of no pain in the head. The mercurial ointment was omitted.

On the 5th he was tolerably sensible, and excessively peevish — the pupils more active—no pain in the head. A wound on the great toe of the left foot had suppurated, and the absorbents had become inflamed to above the knee. The head symptoms now disappeared very rapidly, and the inflammation of the absorbents became the object of attention. It was treated with salines, and cal. gr. j. pulv. ipec. c. gr. iij. twice daily. On the 11th, the nitrate of silver, in substance, was applied from the knee to near the groin, over the track of the inflamed absorbents. The leg and foot were swollen, red, and very tender to the touch; the pulse was quick, the tongue red, and the bowels loose. An abscess formed in the cellular membrane of the dorsum of the foot, and was opened—the patient was put upon cinchona — and the abscess over the little toe was evacuated—on the 24th he was convalescent—and on the 2d June he was discharged. He was affected with deafness, which he had not before the accident, but he said that he remembered having been thrown from the horse.

CASE 6. *History of Concussion — Anomalous and curious Symptoms referrible to the Brain.*

James Cook, æt. 14, admitted June 4, 1830, under Mr. Hawkins.

Tensive pain across forehead, with a feeling of great weight there—noise of hammering in the head—heaviness of eyes, preventing his seeing perfectly—vacant aspect — vertigo and giddiness on attempting to stand. On making him attempt to walk, which he cannot do without support, his legs totter under him, crossing each other with the toes turned inwards—disposition to nausea—occasional chilliness succeeded by heat. Pulse somewhat hard and full—tongue white, coated—bowels opened by medicine—urine high-coloured.

Just above and behind right ear, a tenderness on firm pressure, and, perhaps, a little more fulness than natural.

One month ago fell from a low two-story window, and struck his side, and the posterior part of the head, where the tenderness is now. Was stunned for ten minutes, when he recovered. On the next day he had pain in the head and swelling, for which he was bled and cupped; leeches were also applied to the side. He recovered, and weakness only remained. Five days ago was seized with the pain in the forehead and other symptoms, without any previous rigor. Says he has felt occasional chills and nausea since the occurrence of the accident, and they have not latterly been worse.

*V. S. brach. ad* ℥xij. *Hyd. Sub., Pulv. Ant.* āā. gr. iij. *in pil.* ij. *stat. H. Senn. Lot. spt.*

5th. Pain in head rather relieved—pupils dilated and sluggish, more especially the left—pulse frequent and full—motions dark and offensive.

*Hirud.* x. *fronti. Glacies capiti raso. Enema Olei ricini.*

On the 6th, he was still better, and the pain in the forehead was much diminished. He complained of much tenderness at the back of the right ear, and was perfectly deaf on that side. The pupils were less dilated, but had altered in toto, for the right was now more affected than the left. The pulse had sunk from 112 to 62. He was ordered calomel and jalap, and the ice was discontinued in the evening. On the 7th the left pupil was again the most dilated; he had little pain in the forehead. The purgation was continued, with cold to the head.

On the 8th, a blister was placed on the back of the neck. The medical man who had attended him, stated that the lad had been suffering from pain in the head and oddness of manner for some time previous to the fall—that one of his brothers had died of disease of the brain—and that the accident was of so little consequence, that no mention was made of it to the parents for two days after its occurrence.

On the 10th he was much better, had no pain in the head, and the pupils were more natural. After considerable pain in the right ear, a certain quantity of matter was suddenly discharged from it with relief. He continued to improve, and on the 15th, he could walk much better, although he still reeled and had an unconquerable disposition to cross his legs. Another blister was placed behind the ear, the purgation was continued, and on the 19th, the blister was again repeated, and the surface dressed with mercurial ointment.

On the 26th he complained of headache, and was again blistered. On the 6th July he could walk by himself with tolerable steadiness; porrigo scutulata had appeared upon the head. This was attacked with the strong acetic acid, and an ointment consisting of unguent. lytt. ʒij., æruginis æris, ℈i., cerat. cetacei, ℥j. This had no effect on the porrigo, which became established fully, but on the 21st of July, he was so far recovered as to be discharged cured.

He had no pain whatever in the head, nor vertigo; could walk and even run, with only a feeling of stiffness; but had lost the hearing of the right ear.

We find we have not room for some interesting cases of scalp wound, which we hoped to have related; they will form the subject of a future report. The first case detailed in the present one, is a tolerably good sample of the symptoms and progress of concussion. Two bleedings and a blister were sufficient to remove the symptoms and effect a perfect and permanent cure. In this hospital we have rarely or never witnessed those frightful cases of concussion, which the works of some authors would lead us to consider as common. To trust to their descriptions, one would imagine that patients were frequently dying in the first stage, and that the second was one of unmeasured and unmitigated violence, only to be put out, if extinguishable at all, by the abstraction of "immense quantities of blood."* From the character of the writers who have made these statements, it is impossible to imagine that they have attempted to describe more than they have actually witnessed. Perhaps these differences of symptoms may admit of explanation in differences of treatment. Mr. Cooper, and, if we recollect aright, Mr. Abernethy, recommend the exhibition of stimuli in the first stage of concussion, and by a natural consequence increase the excitement of the second. The cases of concussion in which stimulants are required are extremely rare, and their

* It is wonderful what immense quantities of blood it is necessary to take away in these cases, in order to keep down the symptoms of phrenitis.—*Cooper's first lines.*

administration, when not absolutely necessary, must be injudicious and injurious. Indeed, if one may judge by cases, the profession appear to be of this way of thinking, for again and again we have seen patients admitted in a state of complete collapse who had been already bled from the arm. They were blooded because they were run over. We do not mean to say that this is advisable treatment, but the practice of this hospital is amply sufficient to prove, that in the great majority of cases the patient will recover from the state of depression, without the employment of measures calculated to increase the subsequent reaction.

It is in fact very rare for individuals to die of the first stage of concussion, unless it be complicated with other extensive mischief, as fracture of the skull and laceration of the brain, extravasation, internal hæmorrhage, or so forth. In such cases stimulation will do no good, it may do harm; but, if the patient is in danger of sinking, we must necessarily have recourse to it.

The perusal of the foregoing cases will suggest many reflections to the professional reader, on the symptoms, treatment, and remoter consequences of concussion of the brain. It is certainly a subject of considerable practical interest.

